# Formal language assessment in low-educated persons with aphasia: can the lesion effect be distinguished from the education effect?

**DOI:** 10.1590/0004-282X-ANP-2020-0475

**Published:** 2022-03-12

**Authors:** Natalia Malagueta de MEDEIROS, Karin Zazo ORTIZ

**Affiliations:** 1 Universidade Federal de São Paulo, Departamento de Fonoaudiologia, São Paulo SP, Brazil. Universidade Federal de São Paulo Departamento de Fonoaudiologia São Paulo SP Brazil

**Keywords:** Aphasia, Language, Education, Afasia, Linguagem, Educação

## Abstract

Background: Language tests are important in the assessment and follow up of people with aphasia (PWA). However, language assessment in the low literacy population is still a challenge. Objective: To investigate whether a formal evaluation of aphasia is able to distinguish the neurological effect from the effect of low educational level in people with post-stroke aphasia. Methods: The sample consisted of a group of 30 aphasic subjects (AG) and a control group (CG) of 36 individuals, both with an educational level of 1-4 years. The Brazilian Montreal-Toulouse Language Assessment battery was applied to all subjects. Results: There were statistically significant differences between the groups in 19 out of the 20 tasks analyzed. Conclusions: These results suggest that formal evaluation procedures are able to detect language disorders resulting from stroke, even in subjects with low educational level.

## RESUMO

Antecedentes: Os testes de linguagem são importantes para a avaliação e o acompanhamento de pacientes afásicos. Apesar disso, a avaliação de linguagem em indivíduos com baixa escolaridade ainda é um desafio. Objetivo: Investigar se a avaliação formal da afasia é capaz de diferenciar o efeito da lesão neurológica *versus* o efeito da baixa escolaridade em pacientes afásicos, acometidos por acidente vascular cerebral (AVC). Métodos: A amostra foi composta de um grupo de 30 sujeitos afásicos (AG) e um grupo controle (CG) de 36 indivíduos, todos com um a quatro anos de escolaridade. A Bateria Montreal-Toulouse de Avaliação da Linguagem foi administrada a todos os participantes. Resultados: Das 20 tarefas analisadas, 19 apresentaram diferenças significativas entre os grupos. Conclusões: Os resultados sugerem que procedimentos formais de avaliação são capazes de identificar as alterações linguísticas ocasionadas por um AVC, também em pacientes com baixa escolaridade.

## INTRODUCTION

Historically, countries with low literacy levels have had to develop research to verify the impact of low literacy on cognitive functioning and have proposed different scores for memory^1^, attention^2^^,^^3^, executive functions^4^ and other cognitive tests^5^^,^^6^. Concerning to language, statistical differences were found in relation to educational levels in normal subjects in the tasks of oral comprehension, reading, written comprehension, naming, lexical retrieval, dictation, written naming of actions^7^ and, in particular, phonological awareness^8^. A previous study also found that when comparing the scores from a normal highly educated population with those of normal people with low educational level there was a false positive result as if people with low educational had a language disorder^9^. Language is a complex cognitive function and defining procedures for assessing populations with low educational levels is complex because of the formal nature of assessments (tests). The implications of different educational levels on aphasia tests could be significant and raise questions on the appropriateness of tests for assessing these individuals^8^. On the other hand, informal assessment is problematic and can result in clinical issues^10^, since an accurate diagnosis is critical for defining steps in the rehabilitation and follow up of people with aphasia (PWA)^11^. One approach for language assessment is the use of validated, standardized tools^12^, but there is a lack of consensus over what is normal or abnormal on these evaluations.

In this respect, previous studies with aphasic populations with low educational level have proposed the use of adjusted scores for the various language functions assessed by these instruments^13^^,^^14^. Language assessment in the low literacy population with neurological injuries is still a challenge and further investigation on whether the lesion effect can be distinguished from the education effect on language impairments in post-stroke aphasic individuals is warranted.

The objective of this study was to determine whether formal evaluation of aphasia (test) is able to distinguish the neurological lesion effect from the effect of low education in post-stroke aphasic individuals.

## METHODS

This comparative analytical study was carried out at the Department of Speech, Language and Hearing Sciences at Universidade Federal de São Paulo, and was approved by the Research Ethics Committee. After receiving full information about the study, written informed consent was obtained from all enrolled subjects.

The sample consisted of a group of 30 PWA (AG) and a control group (CG) of 36 individuals, all with 1-4 years of education and right-handed. The Brazilian Montreal-Toulouse Language Assessment (MTL-Br) battery^15^ was applied to all subjects. The test is the only Brazilian test for assessing aphasia, but normative data are available only for populations with more than 5 years of education^14^. The battery consists of the following subtests: Structured Interview, Automatic Speech, Oral Comprehension, Written Comprehension, Copying, Written Dictation, Repetition, Reading Aloud, Semantic Verbal Fluency, Non-Verbal Praxis, Naming, Object Manipulation by Verbal Command, Phonological Verbal Fluency, Body part recognition and left-right orientation, Written Naming, Oral Text Comprehension, Number Dictation, Reading of Numbers, Written Text Comprehension, and Numerical Calculation.

The inclusion criteria for the AG were: a single stroke to the left-hemisphere and aphasia diagnosis by speech-language therapist. The exclusion criteria were: history of other neurologic or psychiatric conditions, current uncorrected hearing or visual deficits that could negatively impact the language assessment, language disorder or previous school grade repeats.

The CG was formed by applying a health questionnaire, based on which individuals with a history of psychiatric or neurologic illness or current uncorrected hearing or visual deficits that could negatively impact the language assessment, history of previous learning and/or language difficulties, use or history of use of legal or illegal psychotropic drugs and alcohol abuse were excluded.

The raw scores of the language assessment of the AG and CG were compared. The subjects in the CG were relatives and/or companions of the assessed patients.

The groups were compared for age, sex, and education using the Mann-Whitney test. There was no statistically significant difference between the groups for years of education or sex, but a difference was found for age (AG patients were older than CG subjects). The age effect was therefore controlled for by the multivariate analysis of variance (MANOVA) procedure. Analysis of covariance (ANCOVA) was employed to compare the performance of the study groups on the MTL-BR tasks. A probability (p) of less than 0.05 was considered statistically significant for all tests.

## RESULTS

A total of 66 individuals were assessed, comprising 36 in the CG and 30 in the AG. Mean age in the CG was 48.83 years (SD=13.54 years) and mean education was 3.44 years (SD=4.1 years). Mean age in the AG was 65.47 years (SD=9.52 years), while mean education was 3.20 years (SD=1.16 years).

Of the aphasic patients, 11 had mixed aphasia, 5 had anomic aphasia, 4 had transcortical mixed aphasia, 3 had Wernicke’s aphasia, 3 had global aphasia, 2 had transcortical motor aphasia and 2 had transcortical sensory aphasia.

The comparative performances of the two study groups on all tasks of the MTL-Br Battery, controlled for age, are shown in Table 1.

**Table 1.** Comparison of performance by the two groups on the MTL-Br tasks controlled for age.

MTL-BR Task^1^	Group	Mean	SD	Median	ANCOVA adjusted p-value
Structured interview	CG	25.11	1.33	25.50	<0.001*
	AG	14.50	8.90	16.00	
Automatic speech -form	CG	5.81	0.52	6.00	<0.001*
	AG	3.27	2.41	4.00	
Automatic speech -content	CG	5.75	0.55	6.00	<0.001*
	AG	3.13	2.43	3.50	
Oral comprehension -words	CG	4.94	0.23	5.00	<0.001*
	AG	3.43	1.63	4.00	
Oral comprehension -sentences	CG	11.61	1.54	11.50	<0.001*
	AG	6.30	3.31	6.00	
Written comprehension -words	CG	4.39	1.61	5.00	0.002*
	AG	2.43	1.91	3.00	
Written comprehension -sentences	CG	6.33	1.49	6.00	<0.001*
	AG	2.53	2.50	2.00	
Copying	CG	7.44	1.38	8.00	<0.001*
	AG	2.00	2.95	0.00	
Dictation	CG	14.22	5.19	14.50	<0.001*
	AG	3.30	5.31	0.00	
Repetition -words	CG	10.67	0.83	11.00	<0.001*
	AG	5.57	4.44	7.00	
Repetition -sentences	CG	21.78	0.87	22.00	<0.001*
	AG	7.40	8.58	3.00	
Reading aloud -words	CG	9.06	2.37	9.00	<0.001*
	AG	2.97	3.95	1.00	
Reading aloud -sentences	CG	19.58	3.76	21.00	<0.001*
	AG	6.03	7.95	0.00	
Semantic verbal fluency	CG	16.31	4.60	15.50	<0.001*
	AG	3.60	3.40	3.00	
Non-verbal praxis	CG	23.47	1.06	24.00	0.001*
	AG	16.00	8.55	19.00	
Naming -nouns	CG	21.42	2.67	22.00	<0.001*
	AG	10.03	8.99	11.50	
Naming -verbs	CG	5.28	1.49	6.00	<0.001*
	AG	2.73	2.38	3.00	
Object manipulation by verbal command	CG	12.97	5.26	16.00	0.098
	AG	10.10	5.63	12.00	
Phonological verbal fluency	CG	7.53	5.06	7.00	<0.001*
	AG	2.10	3.58	0.00	
Left-right orientation	CG	3.89	0.67	4.00	0.002*
	AG	2.63	1.43	3.00	
Body part recognition	GC	7.39	1.05	8.00	0.037*
	GA	5.83	2.57	6.50	
Written naming -words	CG	12.14	7.80	14.00	<0.001*
	AG	3.67	6.90	0.00	
Written naming -verbs	CG	2.89	2.07	3.00	0.001*
	AG	0.80	1.75	0.00	
Oral text comprehension	CG	5.69	2.54	6.00	<0.001*
	AG	2.00	2.38	1.50	
Number dictation	CG	4.97	1.30	5.00	<0.001*
	AG	1.90	2.23	0.50	
Reading of numbers	CG	5.22	0.64	5.00	<0.001*
	AG	2.43	2.11	2.50	
Written text comprehension	CG	6.28	2.54	7.00	<0.001*
	AG	1.30	2.83	0.00	
Numerical mental calculation	CG	3.03	1.46	3.00	<0.001*
	AG	0.83	1.12	0.00	
Numerical written calculation	CG	2.83	1.95	3.00	<0.001*
	AG	0.33	0.84	0.00	

SD: standard deviation; ANCOVA: analysis of covariance; CG: control group; AG: aphasic group; *Statistically significant value at 5% level (p<0.05).

A statistically significant difference (p<0.001) was found between the two groups on all tasks, except for the “Object Manipulation”, which proved insensitive for differentiating the lesion effect from the education effect.

## DISCUSSION

The data outlined above suggests that language test like the MTL-BR can be used even in PWA with low educational level, since all but the object manipulation task distinguished PWA from normal subjects. The scores of the two groups for the “Object Manipulation” task were similar. The complexity of the mechanisms required to perform this subtest can help explain the results found. Tasks in the MTL-BR involve multimodal stimuli that can facilitate understanding by the subject and their response because they are analyzed based on a number of different processes^9^^,^^16^^,^^17^. In the Object Manipulation task, auditory, proprioceptive, and visual processing are involved. The familiarity with the objects presented and the tangible effect they evoke may have also facilitated the task execution. These factors likely contributed to the two groups performing similarly on the task. In addition, results of a previous study^18^ have shown that there is a ceiling effect on this task in healthy individuals with low educational level.

Differences between groups were evident in all other tasks. Therefore, despite the formal nature of the test^19^^,^^20^, specific deficits in comprehension and production due to brain damage can be identified.

In conclusion, the formal evaluation is able to detect linguistic disorders due to brain injury even in subjects with low levels of education.
